# Loss-of-function mutations affecting a specific *Glycine max *R2R3 MYB transcription factor result in brown hilum and brown seed coats

**DOI:** 10.1186/1471-2229-11-155

**Published:** 2011-11-09

**Authors:** Jason D Gillman, Ashley Tetlow, Jeong-Deong Lee, J Grover Shannon, Kristin Bilyeu

**Affiliations:** 1 USDA-ARS, Plant Genetics Research Unit, 110 Waters Hall, Columbia, MO 65211, USA; 2 University of Missouri, Division of Plant Sciences, 110 Waters Hall, Columbia, MO 65211, USA; 3 Division of Plant Biosciences, Kyungpook National University, Daegu 702-701, Republic of Korea; 4 University of Missouri, Division of Plant Sciences, University of Missouri-Delta Research Center, Portageville, MO 63873, USA

## Abstract

**Background:**

Although modern soybean cultivars feature yellow seed coats, with the only color variation found at the hila, the ancestral condition is black seed coats. Both seed coat and hila coloration are due to the presence of phenylpropanoid pathway derivatives, principally anthocyanins. The genetics of soybean seed coat and hilum coloration were first investigated during the resurgence of genetics during the 1920s, following the rediscovery of Mendel's work. Despite the inclusion of this phenotypic marker into the extensive genetic maps developed for soybean over the last twenty years, the genetic basis behind the phenomenon of brown seed coats (the *R *locus) has remained undetermined until now.

**Results:**

In order to identify the gene responsible for the *r *gene effect (brown hilum or seed coat color), we utilized bulk segregant analysis and identified recombinant lines derived from a population segregating for two phenotypically distinct alleles of the *R *locus. Fine mapping was accelerated through use of a novel, bioinformatically determined set of Simple Sequence Repeat (SSR) markers which allowed us to delimit the genomic region containing the *r *gene to less than 200 kbp, despite the use of a mapping population of only 100 F_6 _lines. Candidate gene analysis identified a loss of function mutation affecting a seed coat-specific expressed R2R3 MYB transcription factor gene (Glyma09g36990) as a strong candidate for the brown hilum phenotype. We observed a near perfect correlation between the mRNA expression levels of the functional *R *gene candidate and an *UDP-glucose:flavonoid 3-O-glucosyltransferase (UF3GT) *gene, which is responsible for the final step in anthocyanin biosynthesis. In contrast, when a null allele of Glyma09g36990 is expressed no upregulation of the *UF3GT *gene was found.

**Conclusions:**

We discovered an allelic series of four loss of function mutations affecting our *R *locus gene candidate. The presence of any one of these mutations was perfectly correlated with the brown seed coat/hilum phenotype in a broadly distributed survey of soybean cultivars, barring the presence of the epistatic dominant *I *allele or gray pubescence, both of which can mask the effect of the *r *allele, resulting in yellow or buff hila. These findings strongly suggest that loss of function for one particular seed coat-expressed R2R3 MYB gene is responsible for the brown seed coat/hilum phenotype in soybean.

## Background

### Domestication of Soybean

Soybean [*Glycine max *(L.) Merr.] is a remarkable plant, producing both high quality oil and protein and is one of the primary row crops in the United States. Although soybean is relatively new to western agriculture, it has been under cultivation for > 3000 years [1,2]. The transition from wild *Glycine soja *to cultivated *Glycine max *was the result of ancient plant breeders/farmers selecting for a large number of domestication-specific traits (photoperiod insensitivity, lack of shattering, lack of lodging, seed size increases, seed set increases, etc.). Dramatic changes in seed oil/protein content and fatty acid composition have apparently also been selected for during domestication, either directly or indirectly [3,4].

### Genetics of soybean seed coloration

The visual appearance of the soybean seed itself has also been altered as a result of domestication: All *Glycine soja *accessions in the USDA GRIN germplasm collection possess black seed coats, whereas the majority of *Glycine max *germplasm (12880/18585 Soybean entries, accessed 06/07/2011) possess yellow seed coats. Although a small market exists for black soybeans, all modern high yielding cultivars feature yellow seed coats, with a range of hila colors present (brown, black, imperfect black, buff, yellow). Cultivars with pale hila are highly prized for natto and tofu production [5]. Because hilum coloration is controlled by a small number of genes [6], this trait is frequently used by breeders as a readily assayed visible marker for the presence of "off-types" in soybean seed lots. Seed coat and hilum color are relatively simple epistatic multi-genic traits, and variation in hilum and seed coat pigmentation appears to be due to the interaction of four independent loci: *Inhibitor *(*I*), *Tawny *(*T*), an unnamed locus termed *R*, and the flower color locus *W1 *[6-8](Table 1). Other loci with minor effects have been described, but these have not been mapped and the genetics have been incompletely discerned [6-8].

**Table 1:** Simplified description of phenotypic effects of three different genetic loci affecting seed coat and hilum colors, adapted from [8].

*Inhibitor*	*Tawny*	*R*	*W1*	Seed coat color	Hilum color	Pubesence	Flower color
*I*	*T*	*R*	*W1/w1*	yellow	gray	tawny	purple/white

*I*	*t*	*R*	*w1*	yellow	yellow	gray	white

*I*	*t*	*R*	*W1*	yellow	gray	gray	purple

*I*	*T*	*r*	*W1*	yellow	yellow	tawny	purple/white

*I*	*t*	*r*	*W1*	yellow	yellow	gray	purple/white

*i^i^*	*T*	*R*	*W1/w1*	yellow	black	tawny	purple/white

***i^i^***	***T***	***r***	***W1/w1***	**yellow**	**brown**	**tawny**	**purple/white**

*i^i^*	*t*	*R*	*W1*	yellow	imperfect black	gray	purple

*i^i^*	*t*	*R/r*	*w1*	yellow	buff	gray	white

*i^i^*	*t*	*r*	*W1/w1*	yellow	buff	gray	purple/white

*i*	*T*	*R*	*W1/w1*	black	black	tawny	purple/white

*i*	*t*	*R*	*W1*	imperfect black	imperfect black	gray	purple

*i*	*t*	*R*	*w1*	buff	buff	gray	white

***i***	***T***	***r***	***W1/w1***	**brown**	**brown**	**tawny**	**purple/white**

i	t	r	W1/w1	buff	buff	gray	purple/white

**Figure 1:**
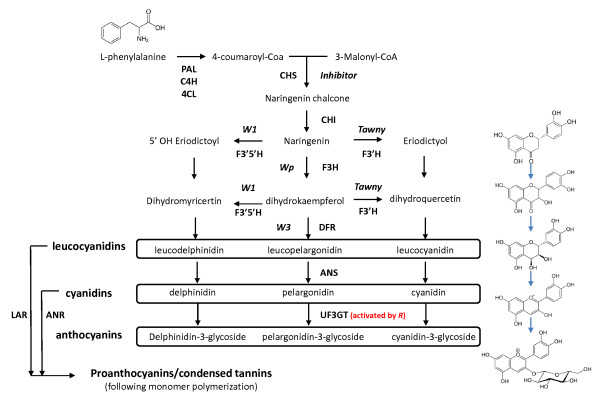
**Simplified representation of the biosynthetic pathway of anthocyanins**. Enzymes are indicated by bold text, intermediates are indicated by plain text, and gene locus designations are in italics. Enzymes are abbreviated as follows: 4-coumarate: CoA ligase (4CL), Anthocyanin Reductase (ANR), Chalcone Synthase (CHS, *Inhibitor *locus), Chalcone Isomerase (CHI), cinnamic acid 4-hydroxylase (C4H), Dihydroxyflavone Reductase (DFR), Flavanone 3-Hydroxylase (F3H, *Wp)*, Flavonoid 5' 3' Hydroxylase (F3'5'H, *W1*), Flavonoid 3' Hydroxylase (F3'H, *Tawny*) Leucoanthocyanidin Reductase (LAR), Phenylalanine Ammonia-Lyase (PAL). The chemical structures to the right of the pathway correspond to eriodictyol, dihydroquercetin, leucocyanidin, cyanidin, and cyanidin-3-glucoside (from top to bottom, respectively).

The compounds responsible for soybean seed coat and hilum color in soybean are derivatives of phenylpropanoid pathway [9-11] (Figure 1). The wild type condition of black seed coats is primarily due to two anthocyanidin glycosides (anthocyanins): cyanidin-3-monoglucoside and delphinidin-3-monoglucoside [10,11]. In lines which feature brown seed coats, only cyanidin is apparently present at maturity [10]. Aside from the cosmetic and aesthetic aspect of coloration, anthocyanins are thought to have diverse human health promoting capabilities [12].

### The action of UDP-glucose:flavonoid 3-O-glucosyltransferase enzymes is a critical step in anthocyanin accumulation

Two anthocyanin glycosides form the predominant colored compounds in black seed coats: cyanidin-3-monoglucoside and delphinidin-3-monoglucoside [10]. These are formed through the action of UDP-glucose:flavonoid 3-O-glucosyltransferase (UF3GT) enzymes, which specifically transfer a glucose moiety from UTP to the 3' position of cyanidin and delphinidin (recently reviewed in [13], Figure 1). This glycosylation is thought to increase the stability and solubility of the cyanidin molecule [14]. In lines with brown seed coats (*r*), cyanidin accumulates, though high levels of proanthocyanidins are also present [10]. Recently, two highly similar co-expressed *UF3GT *genes (Glyma07g30180 and Glyma08g07130) were determined to be expressed in seed coats of black seeded soybean lines, and these genes have been demonstrated to specifically transfer a glucose moiety to the cyanidin molecule at the 3'-hydroxyl group, resulting in the formation of cyanidin-3-glucoside [15].

### The *Inhibitor *locus

Seed coat color is primarily under control of the *Inhibitor *locus, which has at least four classically defined genetic alleles [8], listed here from the most dominant to the least: *I *(largely colorless seeds) >*i^i ^*(color restricted to hilum) >*i^k ^*("saddle;" color in hilum and spreading slightly beyond the hilum) >*i *(seeds completely black). *Inhibitor *acts in a dominant, gain-of-function manner with maternal-effect inheritance, and results in seed coats appearing pale yellow due to the absence of anthocyanins [10]. Both the dominant *Inhibitor *allele (*I*) and the *i^i ^*alleles have been shown to be due to naturally occurring, gene-silencing effects derived from linked but independent Chalcone Synthase (CHS) gene clusters (chromosome 8, LG A2) that generate siRNA which target CHS gene transcripts specifically within the seed coat for degradation [16-22].

### The genetics of soybean hilum coloration

Lines which have the dominant *I *allele can still exhibit some traces of color within the hilum, with the specific hilum coloration due to the allelic status at three other genetic loci: *Tawny*, *R*, and *W1 *[6,8] (Table 1). Hilum tissue is not maternally-derived, in contrast to the seed coat [23]. In lines with the recessive (*i*) allele, seed coat color is brown, imperfect black, buff or black, dependent on the allelic status of the *Tawny, R and W1 *loci (Table 1).

The *Tawny *locus has two pleiotropic effects: homozygosity for the gray (*t*) allele results in gray pubescence at maturity and, in lines carrying the combination of the *i^i ^*allele of the *Inhibitor *locus, a functional *R *gene, and purple flowers (*W1*), seed which feature "imperfect black" hila (Table 1). Alternatively, gray pubescent (*t*) lines carrying the *i^i ^*allele of the *Inhibitor *locus, a functional or nonfunctional *R*, and white flowers (*w1*) produce seed which feature buff hila [8] (Table 1). The phenotypic effects of the recessive allele of *Tawny *have been discerned to be due to loss of function mutations affecting a *flavonoid 3' hydroxylase *gene (Glyma06g21920) [24]. At the chemical level, this is the result of a reduction in the accumulation of anthocyanins within the hilum, and the presence of pelargonidin (Figure 1), which does not accumulate in lines carrying the wild type version of the *Tawny *locus [10].

### The recessive allele of the *R *locus is responsible for brown hilum/seed coats

Another locus, classically termed *R*, also interacts epistatically with the *Tawny *and *Inhibitor *loci (as well as the *W1 *locus) to control hilum and seed coat colors [8] (Table 1). Lines with a functional *Tawny *gene and homozygous for the recessive allele of the *R *locus possess either brown seed coats or brown hilum, dependent on the allelic status of the *Inhibitor *locus (*i *or *i^i ^*respectively). Although the genetics behind this trait were well resolved shortly after the rediscovery of Mendel's work in the 1920s [6], the molecular genetic basis has not been ascertained. Despite this, the ease of phenotyping has resulted in the inclusion of this locus in the development of genetic maps for soybean [25-27].

### Epistasis for genes involved in soybean coloration

Epistatic and pleiotropic interactions are the norm for genes involved in soybean coloration (Table 1). For example, loss of function mutations affecting a *flavonoid 3'5'-hydroxylase *gene (*w1*, *F3'5'H*, Figure 1) have been demonstrated to result in two phenotypes: white flowers and loss of purple pigment in hypocotyls [28]. The allelic status of the *W1 *locus, when combined with the recessive gray allele of the Tawny locus, determines if seed coats or hila are colored "imperfect black" or "buff" (Table 1) [8].

### Approaches to identify the r locus, which results in brown hilum/seed coats

Loss of function mutations affecting a gene involved in the terminal end of the anthocyanin biosynthetic pathway have been suggested as the cause of the recessive brown seed coat/hilum phenotype (Figure 1). Possible candidates have included *UF3GT*, *Anthocyanidin Synthase *(*ANS*) and/or *Dihydroxyflavone Reductase *(*DFR*) genes. However, no correlation has been found between the genomic locations of any *UF3GT*, *DFR *or *ANS *gene and the location of the *R *gene [29]. Alternately, a transcription factor or other regulatory element could be responsible for the brown hilum/seed coat phenomenon. The objective of this work was to identify the specific gene and causative basis behind the phenomenon of brown hilum/seed coat coloration, historically defined as the R locus, in soybean.

## Methods

### RIL population development

The generation of the F_6 _RIL mapping population, derived from a cross between Jake X PI 283327, was previously described [30]. Jake (PI 643912) has tawny pubescence, purple flowers, and shiny yellow seed with black hila (*i^i ^T R W1*)[31]. The brown hila line, PI 283327 has tawny pubescence, purple flowers, and yellow seed with brown hila (*i^i^, T, r, W1*) (USDA GRIN germplasm collection, accessed 06/22/2011 (http://www.ars-grin.gov/npgs/). The reference cultivar Williams 82, for which the genome sequence was determined [32], has tawny pubescence, white flowers and yellow seed with black hila (*i^i^, T, R, w1*) [33].

### Bulk segregant analysis of selected RIL lines

A total of 100 F_6 _RIL lines were selected from a Jake X PI 283327 cross in which segregation for hilum color had occurred (50 possessed black hila, 50 had brown hila) and seed from each were pooled to form two bulks. Only RILs that were definitively black or brown were used in the bulks, with ambiguous or mixed RILs not included. The seeds (1 per RIL) were ground utilizing a coffee grinder to generate a fine powder. The grinder was cleaned thoroughly between grindings. DNA was isolated using a DNeasy Plant Maxi Kit (Qiagen, Inc., Valencia, CA) according to manufacturer's recommendations. Bulk DNA was concentrated using standard ethanol precipitation procedure to yield a final concentration of 3.52 micrograms mL^-1 ^(black bulk) or 2.40 micrograms mL^-1 ^(brown bulk). Bulk DNA was used with Universal Soybean Linkage Panel (USLP) as previously described [34].

### Simple Sequence Repeat (SSR) markers

All SSR primer pairs from within the newly delimited *R *locus region, drawn from a bioinformatically defined list, were also examined for potential utility in fine-mapping [35]. Fine mapping PCR was performed in 20 microliter reactions as previously described [36] and PCR products were separated on 2% agarose gels. Genotypic classes were assigned by visual comparison to PCR reactions using DNA from parental lines. Only those SSR primer pairs which showed obvious, easily scored size polymorphism between the two parents (PI 283327 and Jake) were used in subsequent analysis. SSR primers pairs which displayed polymorphism within the newly defined *R *region, and which could theoretically be used to select for this trait, are listed in Additional File 1.

### DNA isolation, PCR and sequencing of candidate genes from pureline seed

DNA was isolated using a DNeasy plant mini kit (Qiagen), and 5-50 ng of DNA were used in PCR with Ex taq (Takara) with gene specific primers (Additional File 1) under the following conditions: 95°C for 5 minutes, followed by 40 cycles of 95°C for 30 seconds, 59°C for 30 seconds, and at 72°C for 1 minute per 1 kbp of predicted product size. Following PCR, products were examined on a 1% agarose gel by electrophoresis and sent for sequencing at the University of Missouri DNA core facility. Sequence traces were downloaded, imported into Contig Express model of the VectorNTI Advance 11 software (Invitrogen, Carlsbad, CA, USA), assembled and manually evaluated for polymorphisms. Putative polymorphisms were verified by a second, independent PCR and sequencing reaction.

### Selection of diverse lines from the germplasm repository

136 lines were selected for sequencing of the putative *R *gene, drawn either from a previously established list of diverse germplasm [37] or were individually selected from the USDA GRIN germplasm collection (http://www.ars-grin.gov/npgs/) to ensure a broad geographic distribution with a range of hilum and seed coat colors. Certain color classes were only minimally investigated, due to epistatic interactions which precluded novel information (e.g. yellow seed coat with buff hila, see Table 1). A full listing of the 136 lines examined for the allelic status of the *R *gene/Glyma09g36990 is listed in Additional File 2. For a subset of ten lines, all three exons were examined by sequencing (including the 5' UTR, 3'UTR, the 1^st ^intron and the majority of the 2^nd ^intron, although portions of the 2^nd ^intron are highly repetitive AT-rich and recalcitrant to PCR and sequencing). These lines were: PI 84970 (Hokkaido Black, black seed coats), PI 518671 (Williams 82, yellow seed coats, black hila), PI 643912 (Jake, yellow seed coats, black hila), PI 548461 (Improved Pelican, yellow seed coats, brown hila), PI 548389 (Minsoy, yellow seed coats, brown hila), PI 438477 (Fiskeby 840-7-3, yellow seed coats, brown hila), PI 180501 (Strain #18, yellow seed coats, brown hila), PI 283327 (Pingtung Pearl, yellow seed coats, brown hila), PI 240664 (Bilomia No. 3, yellow seed coats, brown hila), PI 567115 B (MARIF 2782, black seed coats). Because all mutations identified were found to affect the 1^st ^or 2^nd ^exons, we elected to only sequence the first and second exons (as well as 5' UTR, the 1^st ^intron, and a portion of the 2^nd ^intron) in the remaining 126 lines.

### qRT-PCR

Expression analysis on seed coat, cotyledon or leaf total RNA (DNAse-treated using Turbo DNase (Ambion, Austin, TX, USA)) was performed as described [38] with minor modifications. The RT-PCR mix was supplemented with 0.2X Titanium Taq polymerase (BD Biosciences, Palo Alto, CA) to improve primer efficiency. Following the reverse transcriptase reaction, amplification was 95°C for 15 min, then 35 cycles of 95°C for 20 seconds, 60°C for 20 seconds, and 72°C for 20 seconds. Primers used in this work are listed in Additional File 1. The reference gene used to normalize data was CONS6 [39] and raw Ct values were first applied to efficiency curves developed for each primer set utilizing Williams 82 genomic DNA, then normalized to the expression of the reference gene and expressed as a percent of CONS6.

Numerous researchers have reported reliable data from qRT-PCR utilizing RNA from mature yellow seed coat tissue. However RT-PCR using RNA derived from brown seed coat tissue was challenging, likely owing to the known effect of interference due to proanthocyanins [10]. The use of a simple PCR Inhibitor removal column (Zymo, Irvine, CA, USA) remedied this difficulty, resulting in acceptable qRT-PCR data derived from mRNA isolated from maturing brown seed coat tissue.

We also investigated CHS7/8 using a primer pair previously described [18]; however the results were highly variable in both cotyledon and seed coat tissues with no significant expression level differences detected between the brown and black seed coat samples (data not shown).

## Results

### Bulk Segregant Analysis

In order to identify the gene responsible for the *r *locus effect (brown hilum or seed coat color), we initially utilized the bulk segregant analysis (BSA) [40] method on RILs from a population derived from the cross of soybean cultivar Jake with the PI 283327 which had segregated for the *R *gene alleles with the USLP array [34]. Although this technique confirmed the previously identified location of the *R *locus [25,26], the extremely broad window identified (data not shown, ~4.2 Mbp, based on the Williams 82 sequence) failed to further delimit the boundaries of the *R *locus.

We then assayed a novel SSR set [35] derived from bioinformatic analysis of the whole genome shotgun sequence (WGSS) for Williams 82 corresponding to the region containing the *R *locus. The use of DNA from the two bulks with polymorphic markers allowed us to refine the *R *region to ~1.35 Mbps as tightly linked to the locus responsible for brown hila (Table 2).

**Table 2:** Polymorphic markers used in BSA to identify lines featuring recombination near the *R *locus.

Polymorphic marker	Complete linkage using BSA?	Recombinant RIL identified	Gm09 marker start position	Gm09 marker end position
BARCSOYSSR_09_1445	no	numerous	41776033	41776086

BARCSOYSSR_09_1453	no	numerous	41890948	41891009

BARCSOYSSR_09_1458	no	numerous	41990780	41990801

**BARCSOYSSR_09_1475**	**yes**	yes	42289944	42290027

**BARCSOYSSR_09_1489**	**yes**	yes	42537113	42537168

**BARCSOYSSR_09_1492**	**yes**	**no**	**42548681**	**42548700**

**BARCSOYSSR_09_1501**	**yes**	**no**	**42635803**	**42635834**

**BARCSOYSSR_09_1504**	**yes**	**no**	**42678917**	**42678946**

**BARCSOYSSR_09_1506**	**yes**	yes	42730901	42730932

**BARCSOYSSR_09_1512**	**yes**	yes	42848842	42848903

**BARCSOYSSR_09_1514**	**yes**	yes	42871791	42871814

**BARCSOYSSR_09_1535**	**yes**	yes	43185760	43185810

BARCSOYSSR_09_1563	no	numerous	43586131	43586156

BARCSOYSSR_09_1566	no	numerous	43644804	43644847

All markers indicated are drawn from the recently described list of Simple Sequence Repeat markers determined by bioinformatics analysis [35] of the Williams 82 whole genome shotgun sequence [32].

### Identification of lines featuring recombination events within the delimited *R *region

**Figure 2:**
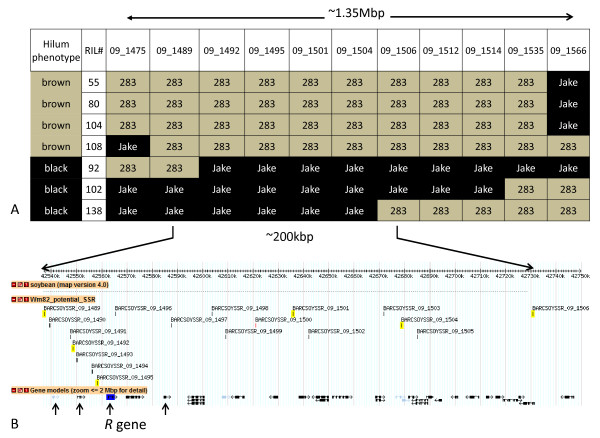
**Diagram of genetic mapping of the gene responsible for brown hilum in PI 283327**. **2A**: Diagram depicting the phenotype and allelic status of SSR markers within F_6 _RIL lines used to fine map the locus responsible for brown hilum color in soybean cultivar PI 283327. **2B**: Screen capture of generic genome browser version 1.71, displaying the region identified which contained the locus responsible for the brown hilum color in soybean cultivar PI 283327(http://www.soybase.org, accessed 03-15-2011). Arrows indicate the location of the four candidate R2R3 MYB transcription factor genes. The genomic location of the only R2R3 MYB gene expressed in seed coats, which features a deletion from within exon 2 (C377-) in the brown hilum line (PI 283327) is indicated.

Three primer pairs from the novel SSR set (BARCSOYSSR 09_1475, 09_1501 and 09_1566 were examined for all 100 RIL lines. For the majority, the hilum color phenotype was correlated with the expected parental polymorphic band. We also observed seven individual RILs which possessed recombination events within the region identified on chromosome Gm09/LG K (Figure 2A). We examined these seven RILs using all novel polymorphic SSRs markers within this region, and compared the marker genotype to the RIL phenotype (Table 2 Figure 2A). Our methodology allowed us to fine-map the location of the *R *gene to a predicted region of less than 200 kbp with only 100 RIL lines. This region in Williams 82 contains 23 predicted open reading frames, with another 3 genes annotated as pseudogenes (Figure 2B).

### Identification of four R2R3 MYB genes as candidates for the *R *locus

BLAST searches using the 26 candidate genes were performed against NCBI (http://www.ncbi.nlm.nih.gov/) and TAIR (http://www.arabidopsis.org) databases to search for candidate genes. BLAST searches revealed four tandem genes which featured homology to the R2R3 MYB transcription factor gene family: Glyma09g36970, Glyma09g36980, 09g36990 and Glyma09g37010. R2R3 MYB genes have been shown to control flux through the phenylpropanoid pathway, and mutants in multiple species are associated with changes in fruit, flower and/or seed color (recently reviewed in [41]). These four tandem R2R3 MYB genes are highly similar (~80-90% nucleotide identity, excluding presumed intronic sequence) and may have arisen due to a tandem gene amplification event(s). Strikingly, none of these genes appears to have been identified in recent seed focused studies using RNAseq methods [42,43].

### Expression analysis of R2R3 gene candidates

**Figure 3:**
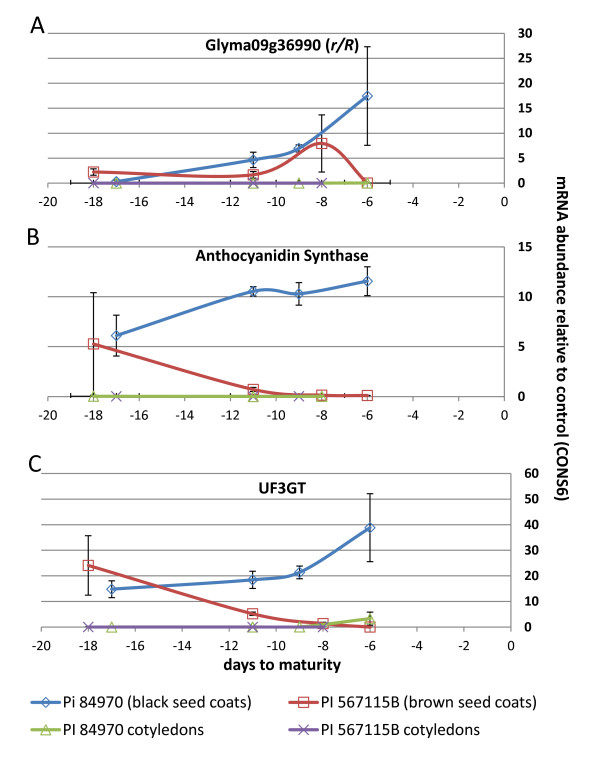
**Quantitative RT-PCR of RNA isolated from seed coat and cotyledon tissue at four stages of development**. Each data point represents the average gene expression for two biological replicates, with three technical replicates for each biological replicate. Vertical bars represent one standard deviation. X-axis indicates days prior to seed maturity. Y-axis indicates gene expression relative to CONS6. **3A: **qRT-PCR of *R *gene candidate Glyma09g36990, expressed as a relative measure of CONS6. **3B: **qRT-PCR of anthocyanidin synthase gene expression (*ANS*, non-gene specific), relative to CONS6. **3C: **qRT-PCR of *UDP-glucose: flavonoid 3-O-glucosyltransferase *(*UF3GT*, Glyma08g07130) gene expression, relative to CONS6.

Because soybean hilum tissue is extremely small and difficult to accurately dissect from seeds in non-pigmented stages, we utilized a large seeded soybean line with brown seed coats (PI 567115 B) and a large seeded line with black seed coats (PI 84970) to examine mRNA expression. In order to assess whether a subset of these four tandem genes were pseudogenes and/or expressed in seed coat tissue (Glyma09g36970 is annotated as a pseudogene in the current whole genome shotgun sequence build), we utilized qRT-PCR. Only one of these candidate R2R3 MYB genes, Glyma09g36990, was expressed in any of the tissues examined (leaf, seed cotyledons, and seed coats). Gene transcripts from Glyma09g36990 were present in the seed coats of both a brown seeded and a black seeded cultivar. However, this gene was not expressed in either cotyledon tissue (Figure 3A) or in leaves (data not shown). It is not clear if the other three R2R3 MYB genes in the cluster are expressed in other tissues. Nor is the role these genes play in soybean physiology known, if any.

Curiously, the Williams 82 Glyma09g36990 gene model was predicted to possess four exons, in contrast to the canonical 3 exons identified for authentic R2R3 MYB transcription factor genes [44,45]. To characterize the authentic expressed sequence, RT-PCR was used to analyze full length cDNA for comparison to the reference Williams 82 gene model. The authentic gene is slightly larger than that the predicted Glyma09g36690 gene model and possesses three exons (Additional File 3), in concordance with that reported for other R2R3 MYB genes [44,45].

### Analysis of Glyma09g36990 for potential causative polymorphisms

**Figure 4:**
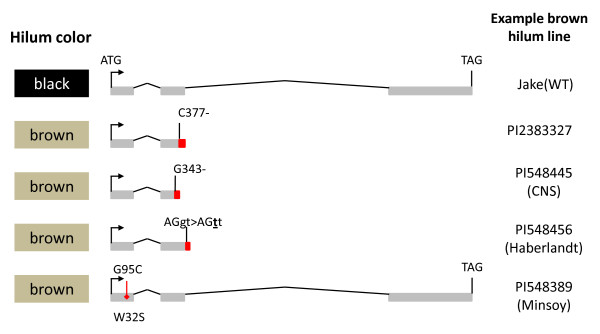
**Genetic alleles of the *R *locus/Glyma09g36990 gene**. Summary of four loss of function alleles identified from 136 soybean cultivars, with one example of commonly used soybean accessions listed. The full list of cultivars examined, and allelic status, is listed in Additional File 2.

PCR and Sanger sequencing of exons (and partial intronic sequence) was used to evaluate the Glyma09g36990 gene for polymorphisms in a selection of lines: Jake (black hilum), PI 283327 (brown hilum), Williams 82 (black hilum), PI 84970 (black seed coats) and PI 567115 B (brown seed coats). We discovered a single-base deletion within exon 2 in PI 283327 and PI 567115 B that results in a frameshift mutation (C377-, relative to the start codon) (Figure 4, details in Additional File 3). The open reading frame for Glyma09g36990 was allelic between Williams 82, Jake and PI 84970.

We then elected to examine a broad geographic distribution of lines (136 in total, Additional File 2) from the available soybean germplasm corresponding to all of the known seed coat and hilum color classes. From this pool, we identified three additional presumed loss of function mutations: G343-, resulting in frameshift; G95C TGG > TCG (W32S) missense in conserved residue; AGgt > AGtt (g404t) disrupts conserved mRNA splice recognition site (Figure 4, further details in Additional File 3).

In all cases where we observed an intact open reading frame, we noted the phenotype of imperfect black hilum (*i^i ^R t W1*), buff hilum (*i^i ^R t w1*), black hilum (*i^i ^R T*) or black seed coat (*i R T*), dependent on the allelic status of the *Inhibitor *and *Tawny *loci (Additional File 2). Any of these four loss of function alleles resulted in either brown hilum (*i^i ^r T*), brown seed coat *(i r T*) or buff hila (*i^i ^r t*). In all cases, we observed a perfect association between the presence of one of the four loss of function alleles and brown hilum or brown seed coats, barring the presence of the epistatic dominant *I *allele or gray pubescence, both of which can mask the effect of the *r *allele, resulting in yellow or buff hila (Additional File 2). These epistatic interactions (and masking in the case of *Inhibitor*) are due to the placement of the step affected by the R2R3 MYB gene at the terminal end of the anthocyanin biosynthesis pathway (Figure 1). Any one of the loss of function mutations affecting the *R *gene are necessary and sufficient for brown seed coat and/or hilum coloration. However, the phenotypic effect can be masked or modulated by the presence of certain alleles of the Inhibitor and Tawny loci (Table 1 Additional File 2).

### Time-course of mRNA expression for Glyma09g36990 and two phenylpropanoid biosynthetic enzymes

If the candidate *R *gene is controlling expression of a gene which forms a rate limited step in anthocyanin production, we hypothesized that a correlation would exist between 1) *R *gene expression levels, 2) the appearance of color compounds, and 3) the expression of *ANS *and/or *UF3GT *genes in developing seed coats. We examined a time course of seed coat and seed cotyledons by qRT-PCR (Figure 3) for expression of three genes: the *R *gene candidate, *ANS*, and *UF3GT*. Seed coats from the large seeded line with brown seed coats (PI 567115 B) and one with black seed coats (PI 84970) were investigated for quantitation of steady state transcripts. We selected four time-points corresponding to the development of pigmentation during seed growth and maturation for PI 84970 (black seed coats) and PI 567115 B (brown seed coats) (Additional File 4).

Although there are apparently two *UF3GT *genes expressed in seed coats in soybean (Glyma07g30180 and Glyma08g07130), only one of these genes (Glyma08g07130) is not expressed in cotyledon tissue [15]. We elected to focus on this gene for qRT-PCR, as we noted a virtual absence of *ANS *or *R *gene expression in cotyledons (Figure 3A and 3B).

We observed a near-perfect coefficient of correlation (R^2 ^= 0.96) between the level of expression (relative to an internal control CONS6) of the putative *R *gene and a *UF3GT *gene (Glyma08g07130) (Figure 3A and 3C). In contrast, we observed a weak correlation between expression of the *R *gene and *ANS *gene expression (R^2 ^= 0.66) in the black seed coat line (Figure 3A and 3B). In the brown seeded line PI 567115 B, no significant correlation was found between *R *gene expression levels and either *ANS *or *UF3GT *expression levels (Figure 3A-C). During early and mid-development stages *R *gene expression is similar in both black and brown seed coat lines, though *R *gene expression declined during the last stages of development of the brown seeded line, in contrast to the high expression noted for the black seed coat lines (Figure 3A). In striking contrast to the increase in expression of *ANS *and *UF3GT *during seed coat maturation of the black seed coat line, only negligible *ANS *and *UF3GT *expression was observed in the brown seed coat line as seeds approached maturity (Figure 3B and 3C).

These findings confirmed our hypothesis that loss of function mutations within Glyma09g36690, an R2R3 MYB gene, are correlated with reduced expression of a *UF3GT *gene and *ANS *genes and with the brown hilum/seed coat phenotype. It remains to future work to determine the specific DNA sequence targeted by the soybean R2R3 MYB *R *gene product and its specific interactions in complexes with basic-helix-loop-helix (bHLH) transcription factors and WD40 proteins. It is unclear if the R gene product acts to promote transcriptional activation of both *ANS *and *UF3GT *genes, or if activation of *ANS *gene expression is due to an indirect effect.

## Discussion

Understanding the genetic factors controlling the accumulation of different colored, easily categorized exterior pigments (both plant and animal produced) became one of earliest models for the confirmation and expansion of Mendel's laws of inheritance. Indeed, modern genetics owes a strong debt to the white color trait in pea, which was exploited by Mendel in the original determination of basic genetic theory [46]. The specific genetic cause of the white flower phenotype in pea has been ascertained as a point mutation disrupting a splice site within a bHLH transcription factor [47]. The study of variation in seed coat colors in many plant species has continued to be an area of active research for nearly a century. Over time, a mechanistic understanding of the enzymes responsible for the individual steps involved in pigment formation, the chemistry of the pigments, and also the regulation of those enzymes and pathways by coordinated interaction of transcriptional activators have largely been resolved.

One of the characteristic features of the accumulation of plant pigments that has emerged is the regulation of critical structural genes by R2R3 MYB transcription factors in complexes with bHLH transcription factors and WD40 proteins [48]. R2R3 MYB genes tend to display limited homology (aside from the highly conserved DNA binding region), and the code by which R2R3 MYB genes bind to specific sequences has not been well elucidated [45,48]. These difficulties can complicate phylogenetic analysis and the assignment of genes to paralogous functions. Nevertheless, the soybean *R *gene candidate Glyma09g36990 shows homology to R2R3 MYB genes (Additional File 3). In the past few years a plethora of R2R3 genes have been found which directly impact expression of *UF3GT *and/or phenylpropanoid pathway derived color compound accumulation in seed coats [49], fruits [41,50-52], flowers [50,53,54] and other tissues [55-57]. Aside from the aesthetic appeal of colored compounds, many of these color compounds may have roles as nutraceuticals [12]. Loss of function mutations within R2R3 genes have also been discerned as causative for loss of anthocyanin accumulation in other plant species [57,58]. Although an R2R3 MYB gene(s) would be logical *a priori *candidates for the underlying basis of the *R *locus, the low level of overall homology among R2R3 MYB genes, the presence of at least 448 MYB genes within the soybean genome [59] and the relatively poorly defined genetic map location for the *R *locus [25-27] precluded candidate gene analysis prior to our fine-mapping effort.

Here we used genetic mapping and candidate gene association in a RIL population and a panel of soybean lines with defined coloration (seed coat and hilum, pubescence, and flower) to determine the *R *gene controlling black or brown seed coat in soybean is the R2R3 MYB gene Glyma09g36990. Indirect evidence supports a model in which a functional *R *gene acts to promote transcription of the anthocyanidin late pathway structural genes *U3FGT *as well as *ANS*. These results are consistent with many other instances of a transcriptional regulatory activation control point for genes in the anthocyanidin pathway [41,49-58].

All of the *Glycine soja *accessions in the USDA germplasm collection have black seed coats and thus functional versions of the R gene, while *Glycine max *has both functional and mutant alleles of the *R *gene. Three null alleles of the *R *gene and one allele with a presumed severely deleterious missense mutation were present in our survey of a subset of the soybean germplasm, all of which are correlated with brown hilum or seed coat colors in our survey. Of the lines containing a mutant R gene, the three null alleles had frequencies of ~53%, ~21%, and ~19%, while the missense mutation allele had a frequency of ~6% in our limited survey of 136 divergent lines. This result suggests that multiple independent occurrences of natural mutations from *R *to *r *were selected after soybean domestication but prior to full dispersion of the crop across Asia, since no clear geographical association can be made for any particular allele. The absence of selection pressure for seed coat or hilum color may have allowed broad dispersal of the different alleles. The recently discovered gene for the determinate growth habit in soybean, *dt1*, is an ortholog of the Arabidopsis *terminal flower 1 *gene [37]. Coincidentally, the *dt1 *gene also has an identified functional allele as well as four mutant alleles associated with a determinate growth phenotype. The mutant *dt1 *alleles are present only in *Glycine max*, but these alleles appear to have been undergoing selection pressure at early stages of soybean landrace radiation [37].

Future work may involve targeted overexpression of R2R3 MYB gene in various cotyledon, seed coat and other tissues in soybean. Because the R gene appears to be exquisitely limited in expression to seed coats, overexpression of this gene in other tissues may result in accumulation of anthocyanins in tissues which lack visible pigments, such as seed cotyledons. Potentially, expressing this R2R3 MYB gene under control of a seed storage protein promoter could increase the anthocyanin content of soybean seeds, in contrast to the wild type restriction of anthocyanins to seed coats. Though hypothetical, this may represent a viable, alternate means to visually select for transgene integration and/or a visual means to assist in containment of transgenic lines.

## Conclusions

We performed bulk segregant analysis (BSA) [40] on a F_6_-RIL population which had segregated for hilum color [30], derived from a cross between a commercial cultivar with black hila (Jake) and a plant introduction line with brown hila (PI 283327). We utilized a novel set of bioinformatically derived SSR markers [35] to fine map the *R *gene to less than 200 kilobasepairs, despite using a RIL population of less than 100 individual F_6 _lines. Analysis of the Williams 82 whole genome shotgun sequence [32] corresponding to this region revealed four tandem R2R3 MYB genes as likely candidates for the authentic *R *gene. R2R3 MYB transcription factors are one of the largest transcription factor families in plants [41,44], and specific R2R3 genes have been identified in a number of species which activate phenylpropanoid biosynthetic genes [13,29,41,50,54,56,60,61]. Only one of the four candidate R2R3 MYB transcription factor genes (Glyma09g36990) in the genomic region containing *R *proved to be expressed in any of the tissues we examined. The seed-coat specific expression of the functional version of this gene was strongly correlated with the level of expression of a *UF3GT *gene (Glyma08g07130), which encodes a gene product that carries out the final step in anthocyanin biosynthesis [15]. We discovered an allelic series of loss of function mutations affecting our R2R3 gene candidate, and the presence of any of the four loss of function mutations was perfectly correlated with the brown seed coat/hilum phenotype in a broad distribution of soybean cultivars divergent in seed coat, hilum and flower color. These findings strongly suggest that loss of function for this particular R2R3 MYB gene is responsible for the brown seed coat/hilum phenotype in soybean. The presence of multiple independent alleles suggests that this gene was selected during domestication either directly for brown coloration or indirectly for pale hilum colors (due to its epistatic effects with *Inhibitor *and *Tawny*).

## Abbreviations used

4CL: 4-coumarate: CoA ligase; ANR: Anthocyanin Reductase; BSA: Bulk Segregant Analysis; CHS: Chalcone Synthase; CHI: Chalcone Isomerase; C4H: cinnamic acid 4-hydroxylase; DFR: Dihydroxyflavone Reductase; F3H: Flavanone 3-Hydroxylase; F3'5'H: Flavonoid 5' 3' Hydroxylase; F3'H: Flavonoid 3' Hydroxylase; LAR: Leucoanthocyanidin Reductase; PAL: Phenylalanine Ammonia-Lyase; PI: Plant Introduction line; RIL: Recombinant Inbred Line; SSR: Simple Sequence Repeat; USLP: Universal Soybean Linkage Panel.

## Authors' contributions

JDG conceived of the experiments, authored the manuscript, selected lines for analysis, isolated DNA from lines, performed PCR, RT-PCR, cloning, bulk segregant analysis, SSR genotyping, sequencing reactions and data analysis. AT performed DNA isolation, SSR genotyping, plant growth and maintenance, and seed coat and hilum color phenotyping. KB also conceived of the experiments, performed qRT-PCR, performed data analysis, and also authored the manuscript. JDL and JGS developed the F_6 _RIL population used for bulk segregant analysis. All authors reviewed and approved the manuscript.

## Supplementary Material

Additional file 1 **List of primers used in this work**. Excel format file containing all primers used in cloning the R locus. Click here for file

Additional file 2 **Summary of phenotypic data and allelic status for Glyma09g36990 for 136 selected soybean accessions**. Excel format file containing seedcoat, hilum and flower phenotypic information and R gene allelic status for 136 selected soybean accessions. Click here for file

Additional file 3 **Sequence details of Glyma09g36990, the gene responsible for the r locus**. Word file containing cloned gene model, details of mutations identified and alignment of *R *gene candidate, Glyma09g36990, with four R2R3 MYB genes known to control *UF3GT *expression and/or anthocyanin accumulation in other species. Click here for file

Additional file 4 **Images of seeds selected for quantitative RT-PCR**. Images of intact seeds used for qRT-PCR time course of a brown (PI 567115 B) and a black seeded (PI 84970) cultivar. Click here for file
