# Supplementary material for: Loss-of-function mutations affecting a specific Glycine max R2R3 MYB transcription factor result in brown hilum and brown seed coats
Source: BMC Plant Biol. 2011 Nov 9;11:155. doi: 10.1186/1471-2229-11-155 (PMC3229458; doi:10.1186/1471-2229-11-155)
Supplement: Additional file 3 — Sequence details of Glyma09g36990, the gene responsible for the r locus. Word file containing cloned gene model, details of mutations identified and alignment of R gene candidate, Glyma09g36990, with four R2R3 MYB genes known to control UF3GT expression and/or anthocyanin accumulation in other species. [file 1471-2229-11-155-S3.DOC]

**Additional File 3**

**Title: Sequence details of Glyma09g36990, the gene responsible for the r locus**

**Description: Word file containing cloned gene model, details of mutations identified and alignment of R gene candidate, Glyma09g36990, with four R2R3 MYB genes known to control UF3GT expression and/or anthocyanin accumulation in other species.**

**Glyma09g36990 phytozome predicted 4-exon gene model**

**(predicted exons in yellow, introns lowercase no color)**

TGAAGGAATTGAGTTATATACGTACACCTGAACAAAATTAATGTAGCAAATTAACTTATAAGCACCTGGTCTC~~ATG~~GAAGGATCATCAGGTGTGAGGAAAGGCACATGGAGTCAAATTGAAGATGATCTTCTCAAAGCTTGCGTGCAACTTTATGGGGAAGGAAATTGGCACCTTGTTCCTAAAAGAGCAGgtgtacttctagttaaattttattttttttttcgttttcttcattgcttgtgattgcaatgatatgatataattacataactatgagatcctcataagttgtaataaataataaacatttttatatcttcttgcgtttggtatatataaacgtagGGTTGAACAGATGCCGCAAGAGTTGTAGATTGAGATGGTTGAACTATCTTAAACCAAATATCAAGCGGGGAGATTTCAGTGAAGATGAAATTGATATGATGATCAGATTGCACAAGCTTTTGGGAAACAGgtttgtatattggccattaattaaatcacactactgagtgtagtgatacatatttagaaattaatatgccttatatgagatatttgaatccatattatattatatgcatgataatacagcttggggtgtactattaactttttccaatatttaaaccaaaaagctagttggttttgtttatgctaagagttatttagagattcaaagaagaacaacaaaacaaatctttgttttcatatatatatatatggaatacaatttcacttttgggaactatttttttacatgagaataattaataataattattagattaaaattaaattaaaatataatataataattaaatctctaaaagatttatcaaacaaatgatatcttaaaatatcattaatttatcatcattgtcataactgttatcatcaccgtcattatgactgttgtcgttgtcactgtcaccaccattgttggatgatagcgacgatgatgacaacaatcatgacgatggtggtggtgacagaggtgaaaatcataatgatttttgcaatgttagtaactactgcaataatgaaggtcatgacagtagaggtggtgatagttgacgacgatgatggtgataacagcgacgattatggtggtgactacagcagtgctcatggtggtgacaacgacaattataattatgattagttaattgaaatattctaggaagtgataattaaaatctttttagatttatttattgtttgattaattttttagagatttgatgatatattaaattttaatttaaattttaaatattttaaaatcaattgtttattatttttaattgatgattattattaattgttctcatataagataatgttctcacaaaattatatatatatatatatatatatatatatatatatatatatatatatatatatatatagacatggacagcataaaggttttatacgatgttatttaattaattagattgtcatttataacattgtttgagttttgtggtaattactctaaaagttgcatgtaaaatgatagagtacatcaaaattaaaagaaaagtgattaatgaacttttcatttttcaatagtattacttcgttaaacaaaaattgtttgtcgatgtagATGGTCCCTGATTGCAGGAAGACTTCCGGGAAGAACCTCAAACGATGTAAAAAATTATTGGAACACCTACGCACGCCGTAAATTACACTCTCACAAGAAAGACAACAACATAGAAAAGCAAGCTAGGGCCAAAACAACCGTGAAACCCCACGAAGTTATAAAGCCTgtacctcgagctttaacaaaaacatccccacggttgcaagGGAAATTCATTAATAGTTCAGAAGTTGGTGTTAGTCATGAAGAAGGTGCAACTTCAATATCAGGGTCTGGGAATTGGTGGGAAACTTTTTTAGATGACAAGGAAGACATTGAAGAAGGTAACAACAACAAATGCTTCTTTGGTGGGGAAGATGGAGCACTTGACCTTTGGGGTGAAGAGCTTAATTCAATTGCTTGTGACTTTCTTACACAAGGTGAAACTTGGAGCGATTTTCTTCTTGACCTAGGGCTAGGAGATTAGTGTGTGGTGTTTGTTTTCACAAGGGACCTCAAATTCTAATATGCAAGTACAAATCAAACTTACCTAACATTTGAGTTTCCTTTGTAAAATATATATATTCTTTACTTTGTATTTGTGAATGTATCTATCTTATCAGTATGTTAAAAAAAAAATTATCTGCTCAGTTATACAGAATGTAAGATTATATCTTAATTACTTTAATTAAATTTAACACATTTAATAACATGAATCAATCTTGGCC

***r* gene loss of function mutations identified (positions are relative to start codon)**

C377- frameshift in exon 2 (truncates open reading frame)

G343- frameshift in exon 2 (truncates open reading frame)

AGgt>AGtt (g404t) disrupts conserved splice site recognition site.

G95C TGG>TCG Trp>Ser change in invariant residue (position indicated in alignment below)

**Glyma09g36690 phytozome predicted cds**

ATGGAAGGATCATCAGGTGTGAGGAAAGGCACATGGAGTCAAATTGAAGATGATCTTCTCAAAGCTTGCGTGCAACTTTATGGGGAAGGAAATTGGCACCTTGTTCCTAAAAGAGCAGGGTTGAACAGATGCCGCAAGAGTTGTAGATTGAGATGGTTGAACTATCTTAAACCAAATATCAAGCGGGGAGATTTCAGTGAAGATGAAATTGATATGATGATCAGATTGCACAAGCTTTTGGGAAACAGATGGTCCCTGATTGCAGGAAGACTTCCGGGAAGAACCTCAAACGATGTAAAAAATTATTGGAACACCTACGCACGCCGTAAATTACACTCTCACAAGAAAGACAACAACATAGAAAAGCAAGCTAGGGCCAAAACAACCGTGAAACCCCACGAAGTTATAAAGCCTGGAAATTCATTAATAGTTCAGAAGTTGGTGTTAGTCATGAAGAAGGTGCAACTTCAATATCAGGGTCTGGGAATTGGTGGGAAACTTTTTTAG

**Corrected intron/exon boundaries for Glyma09g36990 - 3-exon gene model validated by mRNA cloning**

(exons in yellow, introns lowercase no color)

TGAAGGAATTGAGTTATATACGTACACCTGAACAAAATTAATGTAGCAAATTAACTTATAAGCACCTGGTCTCATGGAAGGATCATCAGGTGTGAGGAAAGGCACATGGAGTCAAATTGAAGATGATCTTCTCAAAGCTTGCGTGCAACTTTATGGGGAAGGAAATTGGCACCTTGTTCCTAAAAGAGCAGgtgtacttctagttaaattttattttttttttcgttttcttcattgcttgtgattgcaatgatatgatataattacataactatgagatcctcataagttgtaataaataataaacatttttatatcttcttgcgtttggtatatataaacgtagGGTTGAACAGATGCCGCAAGAGTTGTAGATTGAGATGGTTGAACTATCTTAAACCAAATATCAAGCGGGGAGATTTCAGTGAAGATGAAATTGATATGATGATCAGATTGCACAAGCTTTTGGGAAACAGgtttgtatattggccattaattaaatcacactactgagtgtagtgatacatatttagaaattaatatgccttatatgagatatttgaatccatattatattatatgcatgataatacagcttggggtgtactattaactttttccaatatttaaaccaaaaagctagttggttttgtttatgctaagagttatttagagattcaaagaagaacaacaaaacaaatctttgttttcatatatatatatatggaatacaatttcacttttgggaactatttttttacatgagaataattaataataattattagattaaaattaaattaaaatataatataataattaaatctctaaaagatttatcaaacaaatgatatcttaaaatatcattaatttatcatcattgtcataactgttatcatcaccgtcattatgactgttgtcgttgtcactgtcaccaccattgttggatgatagcgacgatgatgacaacaatcatgacgatggtggtggtgacagaggtgaaaatcataatgatttttgcaatgttagtaactactgcaataatgaaggtcatgacagtagaggtggtgatagttgacgacgatgatggtgataacagcgacgattatggtggtgactacagcagtgctcatggtggtgacaacgacaattataattatgattagttaattgaaatattctaggaagtgataattaaaatctttttagatttatttattgtttgattaattttttagagatttgatgatatattaaattttaatttaaattttaaatattttaaaatcaattgtttattatttttaattgatgattattattaattgttctcatataagataatgttctcacaaaattatatatatatatatatatatatatatatatatatatatatatatatatatatatatagacatggacagcataaaggttttatacgatgttatttaattaattagattgtcatttataacattgtttgagttttgtggtaattactctaaaagttgcatgtaaaatgatagagtacatcaaaattaaaagaaaagtgattaatgaacttttcatttttcaatagtattacttcgttaaacaaaaattgtttgtcgatgtagATGGTCCCTGATTGCAGGAAGACTTCCGGGAAGAACCTCAAACGATGTAAAAAATTATTGGAACACCTACGCACGCCGTAAATTACACTCTCACAAGAAAGACAACAACATAGAAAAGCAAGCTAGGGCCAAAACAACCGTGAAACCCCACGAAGTTATAAAGCCTGTACCTCGAGCTTTAACAAAAACATCCCCACGGTTGCAAGGGAAATTCATTAATAGTTCAGAAGTTGGTGTTAGTCATGAAGAAGGTGCAACTTCAATATCAGGGTCTGGGAATTGGTGGGAAACTTTTTTAGATGACAAGGAAGACATTGAAGAAGGTAACAACAACAAATGCTTCTTTGGTGGGGAAGATGGAGCACTTGACCTTTGGGGTGAAGAGCTTAATTCAATTGCTTGTGACTTTCTTACACAAGGTGAAACTTGGAGCGATTTTCTTCTTGACCTAGGGCTAGGAGATTAGTGTGTGGTGTTTGTTTTCACAAGGGACCTCAAATTCTAATATGCAAGTACAAATCAAACTTACCTAACATTTGAGTTTCCTTTGTAAAATATATATATTCTTTACTTTGTATTTGTGAATGTATCTATCTTATCAGTATGTTAAAAAAAAAATTATCTGCTCAGTTATACAGAATGTAAGATTATATCTTAATTACTTTAATTAAATTTAACACATTTAATAACATGAATCAATCTTGGCC

***r* gene loss of function mutations identified (positions are relative to start codon)**

C377- frameshift in exon 2 (truncates open reading frame)

G343- frameshift in exon 2 (truncates open reading frame)

AGgt>AGtt (g404t) disrupts conserved splice site recognition site.

G95C TGG>TCG Trp>Ser change in invariant residue (position indicated in alignment below)

**Glyma09g36990 coding sequence determined experimentally**

**(exons indicated by different highlight colors, placement of loss of function mutations indicated by highlight)**

ATGGAAGGATCATCAGGTGTGAGGAAAGGCACATGGAGTCAAATTGAAGATGATCTTCTCAAAGCTTGCGTGCAACTTTATGGGGAAGGAAATTGGCACCTTGTTCCTAAAAGAGCAGGGTTGAACAGATGCCGCAAGAGTTGTAGATTGAGATGGTTGAACTATCTTAAACCAAATATCAAGCGGGGAGATTTCAGTGAAGATGAAATTGATATGATGATCAGATTGCACAAGCTTTTGGGAAACAGATGGTCCCTGATTGCAGGAAGACTTCCGGGAAGAACCTCAAACGATGTAAAAAATTATTGGAACACCTACGCACGCCGTAAATTACACTCTCACAAGAAAGACAACAACATAGAAAAGCAAGCTAGGGCCAAAACAACCGTGAAACCCCACGAAGTTATAAAGCCTGTACCTCGAGCTTTAACAAAAACATCCCCACGGTTGCAAGGGAAATTCATTAATAGTTCAGAAGTTGGTGTTAGTCATGAAGAAGGCGCAACTTCAATATCAGGGTCTGGGAATTGGTGGGAAACTTTTTTAGATGACAAGGAAGACATTGAAGAAGGTAACAACAACAAATGCTTCTTTGGTGGGGAAGATGGAGCACTTGACCTTTGGGGTGAAGAGCTTAATTCAATTGCTTGTGACTTTCTTACACAAGGTGAAACTTGGAGCGATTTTCTTCTTGACCTAGGGCTAGGAGATTAG

**Glym09g36990 corrected predicted open reading frame**

MEGSSGVRKGTWSQIEDDLLKACVQLYGEGNWHLVPKRAGLNRCRKSCRLRWLNYLKPNIKRGDFSEDEIDMMIRLHKLLGNRWSLIAGRLPGRTSNDVKNYWNTYARRKLHSHKKDNNIEKQARAKTTVKPHEVIKPVPRALTKTSPRLQGKFINSSEVGVSHEEGATSISGSGNWWETFLDDKEDIEEGNNNKCFFGGEDGALDLWGEELNSIACDFLTQGETWSDFLLDLGLGD

**Alignment of predicted protein from corrected Glyma09g36690 gene model with three R2R3 MYB transcription factors demonstrated to control anthocyanin accumulation in other species.**


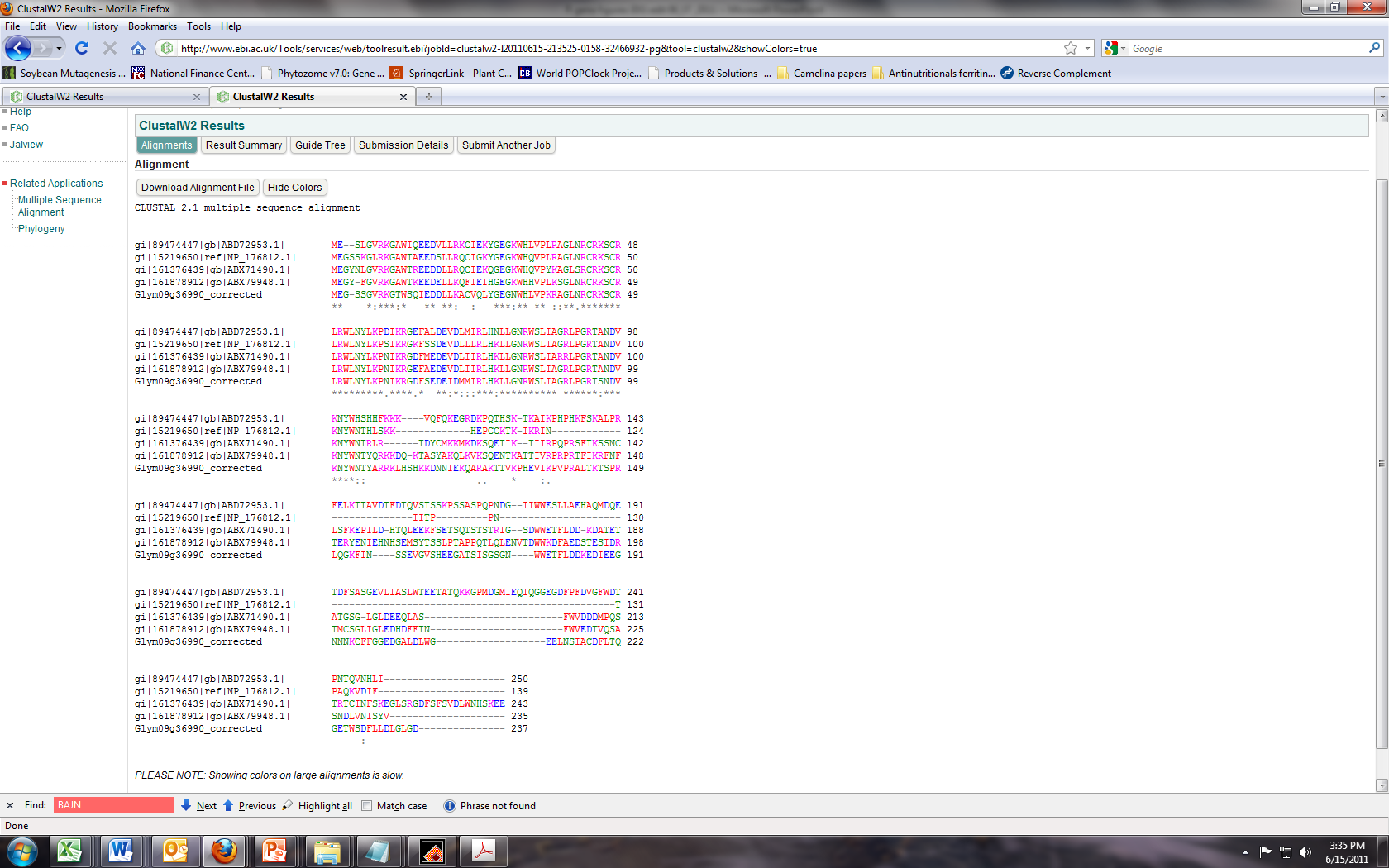


>gi|161376439|gb|ABX71490.1| R2R3 MYB transcription factor 10 [Prunus armeniaca]

MEGYNLGVRKGAWTREEDDLLRQCIEKQGEGKWHQVPYKAGLSRCRKSCRLRWLNYLKPNIKRGDFMEDEVDLIIRLHKLLGNRWSLIARRLPGRTANDVKNYWNTRLRTDYCMKKMKDKSQETIKTIIRPQPRSFTKSSNCLSFKEPILDHTQLEEKFSETSQTSTSTRIGSDWWETFLDDKDATETATGSGLGLDEEQLASFWVDDDMPQSTRTCINFSKEGLSRGDFSFSVDLWNHSKEE

>gi|89474447|gb|ABD72953.1| MybA1 [Vitis vinifera]

MESLGVRKGAWIQEEDVLLRKCIEKYGEGKWHLVPLRAGLNRCRKSCRLRWLNYLKPDIKRGEFALDEVDLMIRLHNLLGNRWSLIAGRLPGRTANDVKNYWHSHHFKKKVQFQKEGRDKPQTHSKTKAIKPHPHKFSKALPRFELKTTAVDTFDTQVSTSSKPSSASPQPNDGIIWWESLLAEHAQMDQETDFSASGEVLIASLWTEETATQKKGPMDGMIEQIQGGEGDFPFDVGFWDTPNTQVNHLI

>gi|161878912|gb|ABX79948.1| R2R3 MYB transcription factor [Fragaria vesca]

MEGYFGVRKGAWTKEEDELLKQFIEIHGEGKWHHVPLKSGLNRCRKSCRLRWLNYLKPNIKRGEFAEDEVDLIIRLHKLLGNRWSLIAGRLPGRTANDVKNYWNTYQRKKDQKTASYAKQLKVKSQENTKATTIVRPRPRTFIKRFNFTERYENIEHNHSEMSYTSSLPTAPPQTLQLENVTDWWKDFAEDSTESIDRTMCSGLIGLEDHDFFTNFWVEDTVQSASNDLVNISYV>gi|15219650|ref|NP_176812.1| transcription factor MYB114 [Arabidopsis thaliana]

MEGSSKGLRKGAWTAEEDSLLRQCIGKYGEGKWHQVPLRAGLNRCRKSCRLRWLNYLKPSIKRGKFSSDEVDLLLRLHKLLGNRWSLIAGRLPGRTANDVKNYWNTHLSKKHEPCCKTKIKRINIITPPNTPAQKVDIF
