# Supplementary material for: Loss-of-function mutations affecting a specific Glycine max R2R3 MYB transcription factor result in brown hilum and brown seed coats
Source: BMC Plant Biol. 2011 Nov 9;11:155. doi: 10.1186/1471-2229-11-155 (PMC3229458; doi:10.1186/1471-2229-11-155)
Supplement: Additional file 4 — Images of seeds selected for quantitative RT-PCR. Images of intact seeds used for qRT-PCR time course of a brown (PI 567115 B) and a black seeded (PI 84970) cultivar. [file 1471-2229-11-155-S4.PPT]

## Slide 1
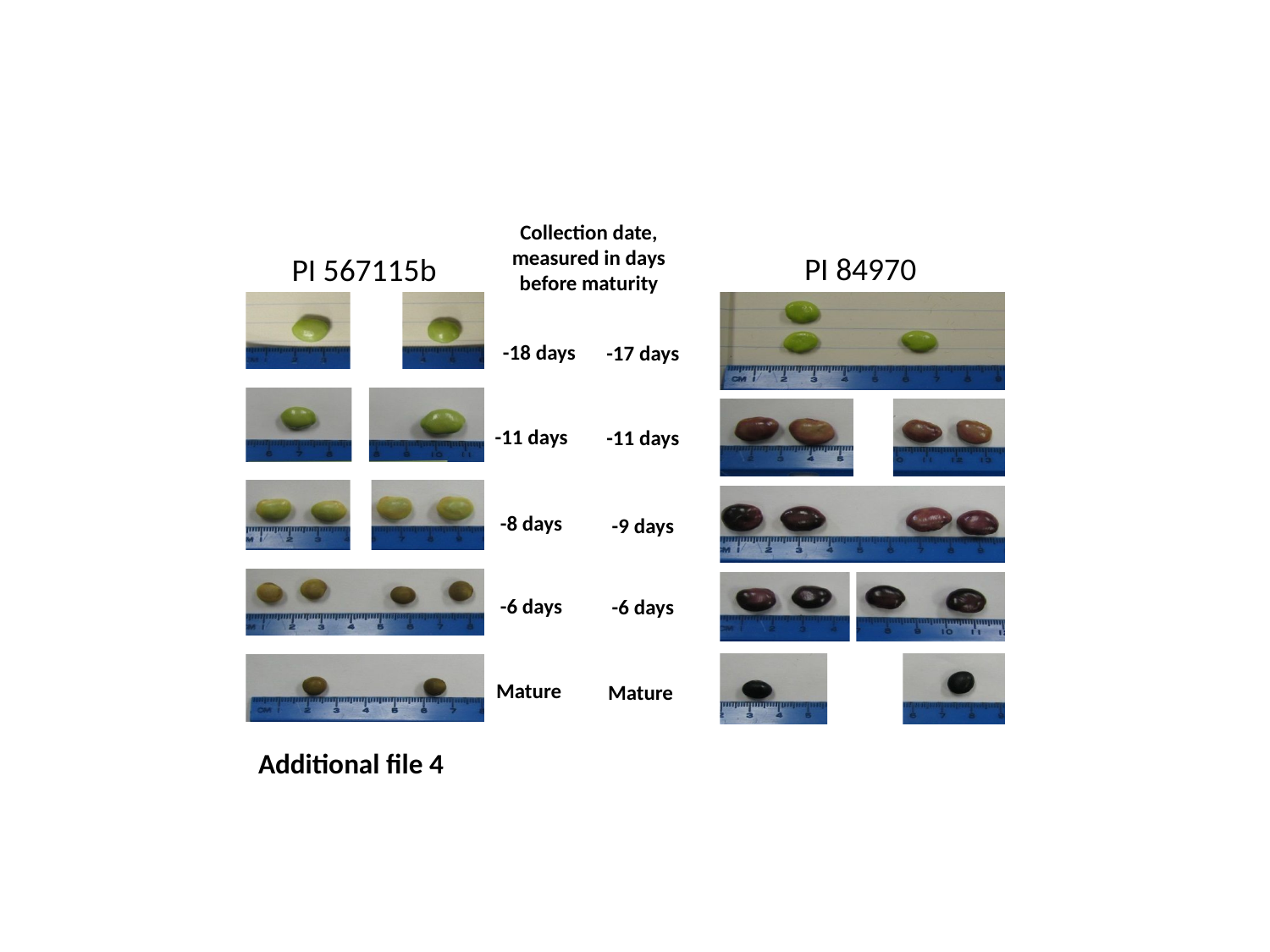

Collection date, measured in days before maturity
PI 84970
PI 567115b
-18 days
-11 days
-8 days
-6 days
Mature
-17 days
-11 days
-9 days
-6 days
Mature
Additional file 4
